# A new one-dimensional Ni^II^ coordination polymer with a two-dimensional supra­molecular architecture

**DOI:** 10.1107/S2056989017000470

**Published:** 2017-01-13

**Authors:** Kai-Long Zhong

**Affiliations:** a Department of Applied Chemistry, Nanjing Polytechnic Institute, Nanjing 210048, People’s Republic of China

**Keywords:** crystal structure, coordination polymer, nickel complex, one-dimensional coordination polymer

## Abstract

A new one-dimensional Ni^II^ coordination polymer of 1,3,5-tris­(imidazol-1-ylmeth­yl)benzene, namely *catena*-poly[[aqua­(sulfato-κ*O*)hemi(μ-ethane-1,2-diol-κ^2^ *O*:*O*′)[μ_3_-1,3,5-tris­(1*H*-imidazol-1-ylmeth­yl)benzene-κ^3^ *N* ^3^,*N* ^3′^,*N* ^3′′^]nickel(II)] ethane-1,2-diol monosolvate monohydrate], {[Ni(SO_4_)(C_18_H_18_N_6_)(C_2_H_6_O_2_)_0.5_(H_2_O)]·C_2_H_6_O_2_·H_2_O}_*n*_, was synthesized and characterized by elemental analysis, IR spectroscopy and single-crystal X-ray diffraction. The Ni^II^ cation is coordinated by three N atoms of three different 1,3,5-tris­(imidazol-1-ylmeth­yl)benzene ligands, one O atom of an ethane-1,2-diol mol­ecule, by a sulfate anion and a water mol­ecule, forming a distorted octa­hedral NiN_3_O_3_ coordination geometry. The tripodal 1,3,5-tris­(imidazol-1-ylmeth­yl)benzene ligands link the Ni^II^ cations, generating metal–organic chains running along the [100] direction. Adjacent chains are further connected by O—H⋯O hydrogen bonds, resulting in a two-dimensional supermolecular architecture running parallel to the (001) plane. Another water mol­ecule and a second ethane-1,2-diol mol­ecule are non-coordinating and are linked to the coordinating sulfate ions *via* O—H⋯O hydrogen bonds.

## Abstract 2

The Ni^II^ cation is coordinated by three N atoms of three different 1,3,5-tris­(imidazol-1-ylmeth­yl)benzene ligands, one O atom of an ethane-1,2-diol mol­ecule, by a sulfate anion and a water mol­ecule, forming a distorted octa­hedral NiN_3_O_3_ coordination geometry. The tripodal 1,3,5-tris­(imidazol-1-ylmeth­yl)benzene ligands link the Ni^II^ cations to generate a metal–organic chain running along [100].

## Chemical context   

In recent years, the self-assembly of coordination polymers and crystal engineering of metal-organic coordination frameworks have attracted great inter­est, owing to their intriguing mol­ecular topologies and the potential applications of these polymers as functional materials (Pan *et al.*, 2004 ▸; Jiang *et al.*, 2011 ▸; Du *et al.*, 2014 ▸). Previously reported studies a major strategy to be the use of multidentate organic ligands and metal ions to construct inorganic–organic hybrid materials through metal–ligand coordination and hydrogen-bonding inter­actions. Imidazole-containing multidentate ligands that contain an aromatic core have received much attention, such as 1,3-bis­(1-imidazol­yl)-5-(imidazol-1-ylmeth­yl)benzene (Fan *et al.*, 2003 ▸), 2,4,6-tris­[4-(imidazol-1-ylmeth­yl)phenyl-1,3,5-triazine (Wan *et al.*, 2004 ▸), 1,3,5-tris­(imidazol-1-ylmeth­yl)-2,4,6-tri­methyl­benzene (Zhao *et al.*, 2004 ▸), 4,4′-bis­(imidazol-1-ylmeth­yl)biphenyl (Carlucci *et al.*, 2008 ▸), 1,3,5-tri(1-imidazol­yl)benzene (Su *et al.*, 2010 ▸), 1,2,4,5-tetra­kis­(imidazol-1-ylmeth­yl)benzene (Hua *et al.*, 2010 ▸) and 1,3,5-tris­(imidazol-1-ylmeth­yl)benzene (Xu *et al.*, 2009 ▸; Zhong, 2014 ▸).

Hydro­thermal (solvothermal) synthesis is an effective method for the construction of new metal–organic coordination polymers because it can provide ideal conditions for crystal growth due to the enhanced transport ability of solvents in superheated systems. In the present work, we carried out the solvothermal reaction between NiSO_4_·6H_2_O and imidazole-containing multidentate ligands, 1,3,5-tris(imidazol-1-ylmeth­yl)benzene (timb) and successfully obtained a new Ni^II^ one-dimensional coordination polymer, {[Ni(SO_4_)(C_18_H_18_N_6_)(C_2_H_6_O_2_)_0.5_(H_2_O)]·C_2_H_6_O_2_·H_2_O}_*n*_, (I). Herein we report its crystal structure and its elemental and IR spectroscopic analysis data.
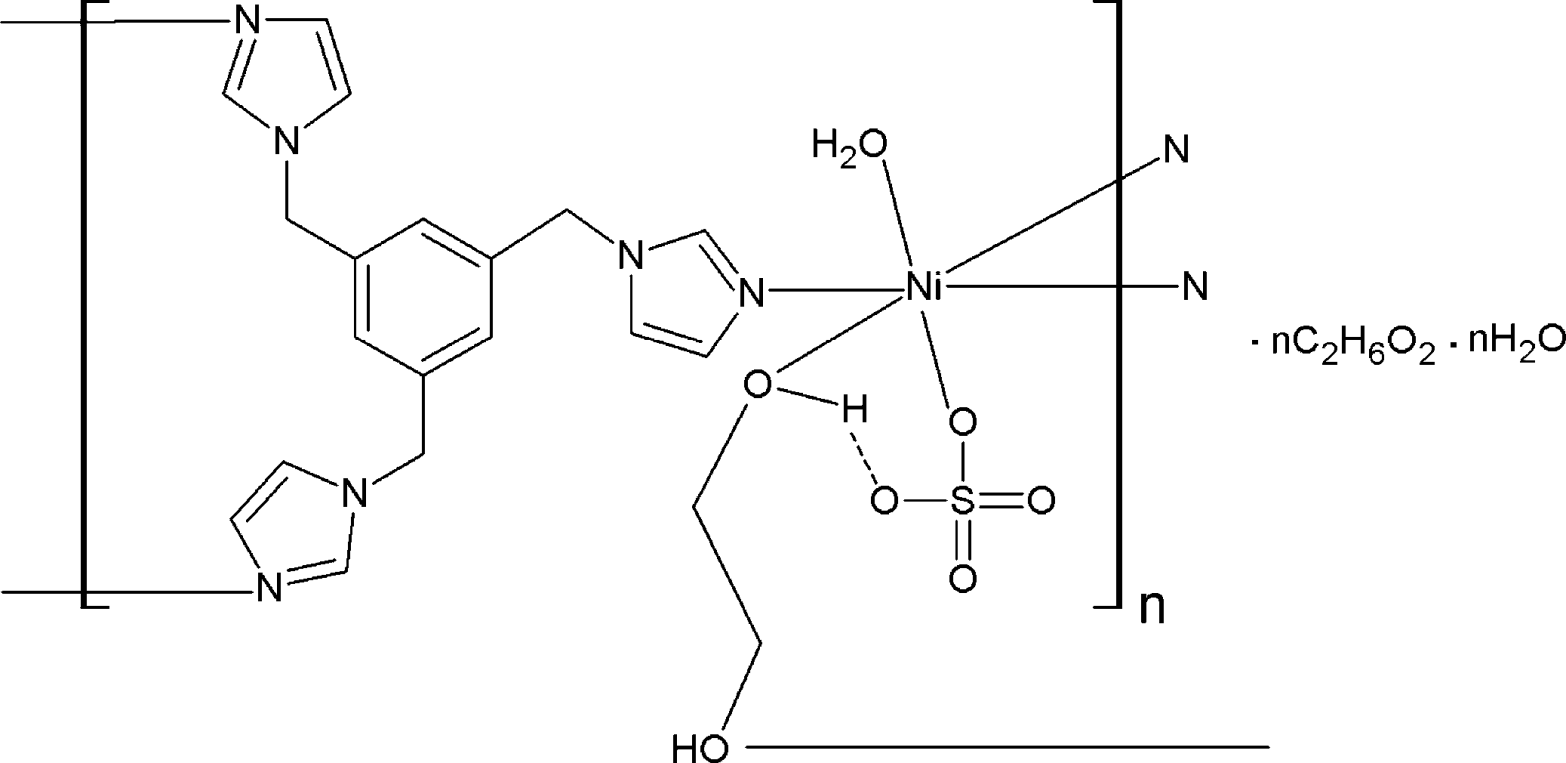


## Structural commentary   

**Figure 1:**
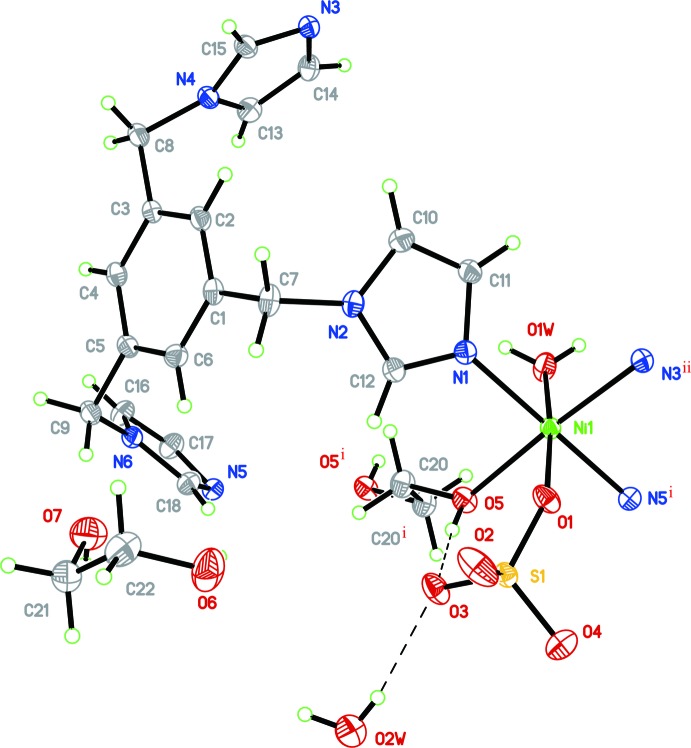
The asymmetric unit of (I), showing the atom-numbering scheme and with displacement ellipsoids drawn at the 25% probability level. All H atoms have been omitted for clarity. [Symmetry codes: (i) −*x*, 1 − *y*, 1 − *z*; (ii) −*x*, −*y*, 1 − *z*.]

The title complex (I) crystallizes in the triclinic space group *P* 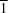 and the asymmetric unit of the structure consists of one Ni^II^ ion, one sulfate anion, one timb ligand, half a coordinating ethane-1,2-diol mol­ecule, one coordinating water mol­ecules, one additional lattice water mol­ecule and one non-coordinating ethane-1,2-diol solvent mol­ecule. As shown in Fig. 1 ▸, each Ni^II^ cation exhibits an irregular octa­hedral NiN_3_O_3_ coordination geometry and is coordinated by three N atoms (N1, N5^i^ and N3^ii^) from three different tripodal timb ligands and three O atoms (O1*W*, O1 and O5) from a coord­inating water mol­ecule, a sulfate anion and a coordinating ethane-1,2-diol mol­ecule, respectively (see Fig. 1 ▸ and Table 1 ▸ for symmetry codes). The Ni—O [2.0904 (12)–2.1458 (12) Å; Table 1 ▸] and Ni—N bond lengths [2.0597 (15)–2.0777 (15) Å] are in accord with corresponding bond lengths found in previously reported Ni^II^ coordination polymers {[Ni(tib)(H_2_O)_2_(SO_4_)]·EtOH·H_2_O}_*n*_ [tib = 1,3,5-tris­(imidazol-1-ylmeth­yl) benzene; Ni—O = 2.0911 (14)–2.1368 (12) Å and Ni—N = 2.0709 (15)–2.0728 (14) Å; Xu *et al.*, 2009 ▸] and [Ni(timpt)_2_](ClO_4_)_2_ [timpt = 2,4,6-tri[4-(imid­azol-1-ylmeth­yl)phen­yl]-1,3,5-triazine; Ni—N = 2.097 (5)–2.151 (4) Å; Wan *et al.*, 2004 ▸].

**Figure 2:**
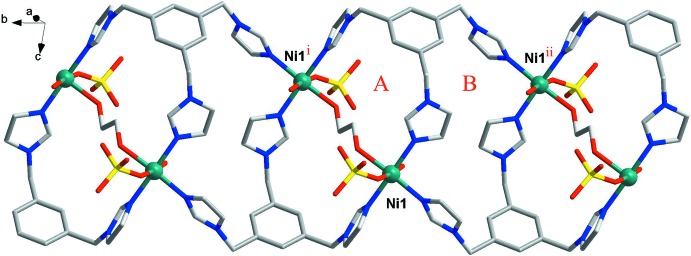
The one-dimensional polymeric chain along the [010] direction. The 17-membered (*A*) and 24-membered (*B*) macrocyclic rings are indicated. [Symmetry codes: (i) −*x*, 1 − *y*, 1 − *z*; (ii) −*x*, −*y*, 1 − *z*.]

Each Ni^II^ atom is coordinated to three individual timb ligands and each timb ligand in turn connects three nickel(II) atoms to generate an infinite laddered chain along the [010] direction (Fig. 2 ▸). Each timb ligand adopts *cis*, *cis*, *cis* substit­uent conformations and coordinates to three Ni^II^ atoms (Ni1, Ni1^i^ and Ni1^ii^), as observed in the Ni compound reported by Xu *et al.* (2009 ▸). The metal–metal distances (Ni⋯Ni) in the above-mentioned chain are 7.1003 (4) Å (Ni1⋯Ni1^i^), 8.7577 (4) Å (Ni1⋯Ni1^ii^) and 11.7296 (6) Å (Ni1^i^⋯Ni^ii^) (see Fig. 2 ▸ for symmetry codes). The three imidazole groups within each timb ligand are inclined to the central benzene ring plane at dihedral angles of 67.60 (6)° (N2/C12/N1/C11/C10), 77.54 (6)° (N4/C15/N3/C14/C13) and 71.75 (6)° (N6/C18/N5/C17/C16), which are different from the values found in a previously reported tib–cadmium compound with the same *cis*, *cis*, *cis* ligand conformations (66.15, 75.58 and 86.33°; Xu *et al.*, 2009 ▸). The three least-square planes of the terminal imidazole rings of the timb ligand are oriented with respect to each other at 56.46 (6)° (N2/C12/N1/C11/C10 and N4/C15/N3/C14/C13), 74.95 (7)° (N2/C12/N1/C11/C10 and N6/C18/N5/C17/C16) and 75.78 (7)° (N4/C15/N3/C14/C13 and N6/C18/N5/C17/C16), respectively.

It can be seen clearly that one 17-membered macrocyclic ring (*A*) and one 24-membered macrocyclic ring (*B*) exist in the above-mentioned chain (see Fig. 2 ▸), which are evidently different from that observed in the previously noted nickel compound {[Ni(tib)(H_2_O)_2_(SO_4_)]·EtOH·(H_2_O)}_*n*_ in which *A* and *B* are 24-membered macrocyclic rings (Xu *et al.*, 2009 ▸).

## Supra­molecular features   

**Figure 3:**
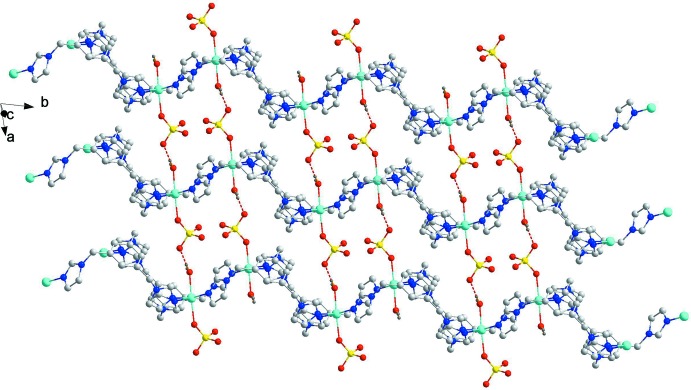
Two-dimensional structure of (I), running parallel to (001) plane. Hydrogen bonds are represented by dashed lines. All lattice water and solvent ethane-1,2-diol mol­ecules have been omitted for clarity.

In the crystal structure of the title compound, the above-mentioned neighbouring chains are further connected to each other by O_water_—H⋯O_sulfate_ hydrogen bonds (O1*W*—H1*WB*⋯O2^iii^), giving rise to a two-dimensional supermolecular structure running parallel to (001) plane (Fig. 3 ▸). Other O—H⋯O hydrogen-bonding inter­actions involve the coordinating water and ethane-1,2-diol mol­ecules, the lattice water mol­ecule, the solvent ethane-1,2-diol mol­ecule and the sulfate anion, *viz.* O1*W*—H1*WA*⋯O6^iii^, O2*W*—H2*WA*⋯O3^iv^, O2*W*—H2*WB*⋯O3, O5—H1*A*⋯O3, and O7—H7*C*⋯O4^iv^ (see Table 2 ▸ for symmetry codes).

## Synthesis and crystallization   

NiSO_4_·6H_2_O (0.1 mmol), 1,3,5-tris­(imidazol-1-ylmeth­yl)benzene (0.1 mmol), water (6 ml) and ethane-1,2-diol (2 ml) were mixed and placed in a thick Pyrex tube, which was sealed and heated to 413 K for 72 h. After cooling to room temperature, blue block-shaped crystals (45% yield, based on Ni) suitable for X-ray analysis were obtained. Elemental analysis calculated for C_21_H_31_N_6_NiO_9_S: C 41.86, H 5.15, N 13.95%; found: C 41.90, H 5.12, N 13.86%. IR (KBr disc, ν, cm^−1^): 3378 (*s*), 1612 (*m*), 1521 (*s*), 1445 (*m*), 1400 (*w*), 1283 (*w*), 1234 (*m*), 1119 (*s*), 1055 (*s*), 963 (*w*), 830 (*m*), 750 (*s*), 661 (*s*), 637 (*m*).

## Refinement   

Crystal data, data collection and structure refinement details are summarized in Table 3 ▸. C-bound H atoms were positioned geom­etrically and allowed to ride on their parent atoms, with C—H = 0.93 or 0.97 Å and *U*_iso_(H) = 1.2*U*_eq_(C). O-bound H atoms of the water and ethane-1,2-diol mol­ecules were either located in difference Fourier maps or placed in calculated positions so as to form a reasonable hydrogen-bonding network, as far as possible. Initially, their positions were refined with tight restraints on the O—H and H⋯H distances [0.82 (1) and 1.35 (1) Å, respectively] in order to ensure a reasonable geometry. They were then constrained to ride on their parent O atoms, with *U*_iso_(H) = 1.5*U*_eq_(O).

## Supplementary Material

Crystal structure: contains datablock(s) I. DOI: 10.1107/S2056989017000470/zl2688sup1.cif

Structure factors: contains datablock(s) I. DOI: 10.1107/S2056989017000470/zl2688Isup2.hkl

CCDC reference: 1470095

Additional supporting information:  crystallographic information; 3D view; checkCIF report

## Figures and Tables

**Table 1:** Selected geometric parameters (Å, °)

Ni1—N5^i^	2.0597 (15)	Ni1—O5	2.0904 (12)
Ni1—N3^ii^	2.0735 (15)	Ni1—O1*W*	2.1048 (12)
Ni1—N1	2.0777 (15)	Ni1—O1	2.1458 (12)
			
N5^i^—Ni1—N3^ii^	89.38 (6)	N1—Ni1—O1*W*	87.11 (5)
N5^i^—Ni1—N1	175.70 (6)	O5—Ni1—O1*W*	89.54 (5)
N3^ii^—Ni1—N1	92.36 (6)	N5^i^—Ni1—O1	93.10 (6)
N5^i^—Ni1—O5	88.70 (6)	N3^ii^—Ni1—O1	90.67 (5)
N3^ii^—Ni1—O5	176.18 (5)	N1—Ni1—O1	90.82 (5)
N1—Ni1—O5	89.79 (5)	O5—Ni1—O1	86.13 (5)
N5^i^—Ni1—O1*W*	88.85 (6)	O1*W*—Ni1—O1	175.21 (5)
N3^ii^—Ni1—O1*W*	93.73 (6)		

Symmetry codes: (i) 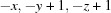; (ii) 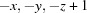.

**Table 2:** Hydrogen-bond geometry (Å, °)

*D*—H⋯*A*	*D*—H	H⋯*A*	*D*⋯*A*	*D*—H⋯*A*
O1*W*—H1*WA*⋯O6^iii^	0.82	1.93	2.743 (2)	172
O1*W*—H1*WB*⋯O2^iii^	0.82	1.88	2.6995 (19)	178
O2*W*—H2*WA*⋯O3^iv^	0.83	2.00	2.827 (2)	176
O2*W*—H2*WB*⋯O3	0.83	2.13	2.928 (2)	163
O5—H1*A*⋯O3	0.81	1.81	2.6168 (18)	169
O7—H7*C*⋯O4^iv^	0.82	2.07	2.884 (3)	174

Symmetry codes: (iii) 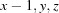; (iv) 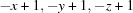.

**Table 3:** Experimental details

Crystal data	
Chemical formula	[Ni(SO_4_)(C_18_H_18_N_6_)(C_2_H_6_O_2_)_0.5_(H_2_O)]·C_2_H_6_O_2_·H_2_O
*M*_r_	602.29
Crystal system, space group	Triclinic, *P* 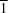
Temperature (K)	223
*a*, *b*, *c* (Å)	8.6910 (4), 11.7296 (5), 13.1200 (6)
α, β, γ (°)	83.922 (1), 77.829 (1), 74.064 (1)
*V* (Å^3^)	1255.53 (10)
*Z*	2
Radiation type	Mo *K*α
μ (mm^−1^)	0.92
Crystal size (mm)	0.30 × 0.25 × 0.20

Data collection	
Diffractometer	Rigaku Mercury CCD
Absorption correction	Multi-scan (*REQAB*; Jacobson, 1998 ▸)
*T*_min_, *T*_max_	0.770, 0.837
No. of measured, independent and observed [*I* > 2σ(*I*)] reflections	10288, 5701, 5102
*R*_int_	0.015
(sin θ/λ)_max_ (Å^−1^)	0.650

Refinement	
*R*[*F* ^2^ > 2σ(*F* ^2^)], *wR*(*F* ^2^), *S*	0.031, 0.077, 1.05
No. of reflections	5701
No. of parameters	346
H-atom treatment	H-atom parameters constrained
Δρ_max_, Δρ_min_ (e Å^−3^)	0.64, −0.35

Computer programs: *CrystalClear* (Rigaku, 2007 ▸), *SHELXT* (Sheldrick, 2015*a* ▸), *SHELXL2014* (Sheldrick, 2015*b* ▸) and *XP* in *SHELXTL* (Sheldrick, 2008 ▸).

## supplementary crystallographic information

### Crystal data

**Table:**

[Ni(SO_4_)(C_18_H_18_N_6_)(C_2_H_6_O_2_)_0.5_(H_2_O)]·C_2_H_6_O_2_·H_2_O	*Z* = 2
*M**_r_* = 602.29	*F*(000) = 630
Triclinic, *P*1	*D*_x_ = 1.593 Mg m^−^^3^
*a* = 8.6910 (4) Å	Mo *K*α radiation, λ = 0.71073 Å
*b* = 11.7296 (5) Å	Cell parameters from 5689 reflections
*c* = 13.1200 (6) Å	θ = 3.6–27.5°
α = 83.922 (1)°	µ = 0.92 mm^−^^1^
β = 77.829 (1)°	*T* = 223 K
γ = 74.064 (1)°	Block, blue
*V* = 1255.53 (10) Å^3^	0.30 × 0.25 × 0.20 mm

### Data collection

**Table:**

Rigaku Mercury CCD diffractometer	5701 independent reflections
Radiation source: fine-focus sealed tube	5102 reflections with *I* > 2σ(*I*)
Detector resolution: 28.5714 pixels mm^-1^	*R*_int_ = 0.015
ω scans	θ_max_ = 27.5°, θ_min_ = 2.5°
Absorption correction: multi-scan (*REQAB*; Jacobson, 1998)	*h* = −11→3
*T*_min_ = 0.770, *T*_max_ = 0.837	*k* = −15→14
10288 measured reflections	*l* = −17→16

### Refinement

**Table:**

Refinement on *F*^2^	0 restraints
Least-squares matrix: full	Hydrogen site location: inferred from neighbouring sites
*R*[*F*^2^ > 2σ(*F*^2^)] = 0.031	H-atom parameters constrained
*wR*(*F*^2^) = 0.077	*w* = 1/[σ^2^(*F*_o_^2^) + (0.0339*P*)^2^ + 0.5937*P*] where *P* = (*F*_o_^2^ + 2*F*_c_^2^)/3
*S* = 1.05	(Δ/σ)_max_ < 0.001
5701 reflections	Δρ_max_ = 0.64 e Å^−^^3^
346 parameters	Δρ_min_ = −0.35 e Å^−^^3^

### Special details

**Table:**

Geometry. All esds (except the esd in the dihedral angle between two l.s. planes) are estimated using the full covariance matrix. The cell esds are taken into account individually in the estimation of esds in distances, angles and torsion angles; correlations between esds in cell parameters are only used when they are defined by crystal symmetry. An approximate (isotropic) treatment of cell esds is used for estimating esds involving l.s. planes.

### Fractional atomic coordinates and isotropic or equivalent isotropic displacement parameters (Å^2^)

**Table:**

	*x*	*y*	*z*	*U*_iso_*/*U*_eq_	
Ni1	0.04969 (2)	0.26468 (2)	0.69398 (2)	0.02264 (7)	
S1	0.39700 (5)	0.31684 (4)	0.73486 (3)	0.02532 (10)	
N1	0.17497 (17)	0.15324 (13)	0.57365 (11)	0.0264 (3)	
N2	0.35427 (17)	0.07352 (13)	0.43798 (11)	0.0272 (3)	
N3	−0.00592 (17)	−0.12373 (13)	0.20513 (11)	0.0275 (3)	
N4	0.12631 (18)	−0.00981 (14)	0.09797 (11)	0.0288 (3)	
N5	0.08774 (18)	0.61960 (13)	0.19341 (11)	0.0280 (3)	
N6	0.28790 (17)	0.48786 (13)	0.10352 (11)	0.0271 (3)	
O1	0.27204 (14)	0.25000 (12)	0.74691 (10)	0.0332 (3)	
O1W	−0.16019 (15)	0.28607 (13)	0.63045 (10)	0.0375 (3)	
H1WA	−0.1485	0.2805	0.5674	0.045*	
H1WB	−0.2450	0.2694	0.6602	0.045*	
O2	0.55747 (16)	0.23427 (14)	0.72399 (15)	0.0551 (5)	
O2W	0.4836 (2)	0.61941 (19)	0.57640 (16)	0.0644 (5)	
H2WA	0.5186	0.6125	0.5127	0.091 (12)*	
H2WB	0.4695	0.5551	0.6032	0.19 (2)*	
O4	0.3645 (2)	0.39103 (17)	0.82364 (12)	0.0585 (5)	
O3	0.38778 (17)	0.39792 (13)	0.63970 (11)	0.0405 (3)	
O5	0.10109 (15)	0.40988 (11)	0.59967 (10)	0.0309 (3)	
H1A	0.1896	0.4142	0.6075	0.066 (8)*	
O7	0.7848 (2)	0.36115 (16)	0.21737 (13)	0.0581 (4)	
H7C	0.7487	0.4328	0.2068	0.087*	
O6	0.8462 (2)	0.27266 (19)	0.42207 (12)	0.0641 (5)	
H6B	0.7551	0.3036	0.4092	0.096*	
C1	0.44203 (19)	0.11243 (16)	0.24838 (13)	0.0270 (4)	
C2	0.3776 (2)	0.05094 (16)	0.19147 (13)	0.0281 (4)	
H2A	0.3650	−0.0240	0.2159	0.034*	
C3	0.3312 (2)	0.10036 (16)	0.09739 (13)	0.0267 (3)	
C4	0.3538 (2)	0.21075 (16)	0.06059 (13)	0.0272 (3)	
H4A	0.3251	0.2435	−0.0026	0.033*	
C5	0.4194 (2)	0.27334 (15)	0.11748 (14)	0.0264 (3)	
C6	0.4632 (2)	0.22389 (16)	0.21129 (14)	0.0290 (4)	
H6A	0.5068	0.2653	0.2496	0.035*	
C7	0.4926 (2)	0.05685 (18)	0.34988 (13)	0.0321 (4)	
H7A	0.5418	−0.0274	0.3423	0.039*	
H7B	0.5740	0.0920	0.3645	0.039*	
C8	0.2624 (2)	0.03201 (19)	0.03467 (14)	0.0349 (4)	
H8A	0.2253	0.0826	−0.0235	0.042*	
H8B	0.3478	−0.0356	0.0064	0.042*	
C9	0.4402 (2)	0.39447 (16)	0.07772 (15)	0.0306 (4)	
H9A	0.5237	0.4113	0.1080	0.037*	
H9B	0.4765	0.3949	0.0025	0.037*	
C10	0.2496 (2)	0.00224 (17)	0.46746 (14)	0.0323 (4)	
H10A	0.2531	−0.0665	0.4367	0.039*	
C11	0.1396 (2)	0.05254 (17)	0.55086 (14)	0.0307 (4)	
H11A	0.0532	0.0232	0.5872	0.037*	
C12	0.3053 (2)	0.16280 (16)	0.50339 (13)	0.0270 (3)	
H12A	0.3563	0.2236	0.4998	0.032*	
C13	−0.0332 (2)	0.05429 (17)	0.12110 (15)	0.0337 (4)	
H13A	−0.0776	0.1313	0.0968	0.040*	
C14	−0.1129 (2)	−0.01670 (17)	0.18614 (14)	0.0329 (4)	
H14A	−0.2239	0.0037	0.2141	0.040*	
C15	0.1375 (2)	−0.11575 (16)	0.15006 (14)	0.0299 (4)	
H15A	0.2336	−0.1758	0.1478	0.036*	
C16	0.1711 (2)	0.52835 (18)	0.04308 (15)	0.0362 (4)	
H16A	0.1747	0.5047	−0.0230	0.043*	
C17	0.0496 (2)	0.60986 (18)	0.09903 (15)	0.0356 (4)	
H17A	−0.0456	0.6528	0.0767	0.043*	
C18	0.2324 (2)	0.54442 (16)	0.19310 (14)	0.0298 (4)	
H18A	0.2886	0.5322	0.2480	0.036*	
C20	0.0760 (2)	0.45061 (18)	0.49659 (15)	0.0349 (4)	
H20A	0.0660	0.3861	0.4600	0.042*	
H20B	0.1682	0.4783	0.4581	0.042*	
C21	0.9401 (3)	0.3387 (2)	0.24521 (18)	0.0498 (5)	
H21A	0.9519	0.4103	0.2700	0.060*	
H21B	1.0250	0.3158	0.1844	0.060*	
C22	0.9574 (3)	0.2421 (2)	0.32855 (19)	0.0533 (6)	
H22A	0.9415	0.1718	0.3039	0.064*	
H22B	1.0673	0.2225	0.3422	0.064*	

### Atomic displacement parameters (Å^2^)

**Table:**

	*U*^11^	*U*^22^	*U*^33^	*U*^12^	*U*^13^	*U*^23^
Ni1	0.02106 (11)	0.02523 (12)	0.02195 (11)	−0.00719 (8)	−0.00350 (8)	−0.00055 (8)
S1	0.02180 (18)	0.0309 (2)	0.0249 (2)	−0.00984 (16)	−0.00678 (15)	0.00368 (16)
N1	0.0254 (7)	0.0282 (8)	0.0252 (7)	−0.0072 (6)	−0.0043 (6)	−0.0008 (6)
N2	0.0267 (7)	0.0305 (8)	0.0220 (7)	−0.0043 (6)	−0.0040 (5)	0.0001 (6)
N3	0.0269 (7)	0.0299 (8)	0.0263 (7)	−0.0102 (6)	−0.0022 (6)	−0.0016 (6)
N4	0.0317 (7)	0.0313 (8)	0.0263 (7)	−0.0151 (6)	−0.0040 (6)	0.0008 (6)
N5	0.0293 (7)	0.0287 (8)	0.0261 (7)	−0.0077 (6)	−0.0049 (6)	−0.0026 (6)
N6	0.0296 (7)	0.0239 (7)	0.0265 (7)	−0.0075 (6)	−0.0019 (6)	−0.0011 (6)
O1	0.0261 (6)	0.0392 (8)	0.0386 (7)	−0.0155 (5)	−0.0128 (5)	0.0111 (6)
O1W	0.0257 (6)	0.0619 (9)	0.0279 (7)	−0.0159 (6)	−0.0043 (5)	−0.0058 (6)
O2	0.0231 (6)	0.0416 (9)	0.0939 (13)	−0.0063 (6)	−0.0090 (7)	0.0162 (9)
O2W	0.0637 (11)	0.0762 (14)	0.0583 (12)	−0.0359 (10)	0.0060 (9)	−0.0144 (10)
O4	0.0787 (12)	0.0705 (12)	0.0375 (8)	−0.0353 (10)	−0.0087 (8)	−0.0141 (8)
O3	0.0393 (7)	0.0474 (9)	0.0394 (8)	−0.0212 (6)	−0.0143 (6)	0.0181 (6)
O5	0.0320 (6)	0.0338 (7)	0.0301 (6)	−0.0112 (5)	−0.0153 (5)	0.0098 (5)
O7	0.0763 (12)	0.0541 (10)	0.0512 (10)	−0.0234 (9)	−0.0231 (9)	0.0046 (8)
O6	0.0594 (10)	0.1007 (15)	0.0345 (8)	−0.0213 (10)	−0.0141 (7)	−0.0020 (9)
C1	0.0224 (7)	0.0313 (9)	0.0232 (8)	−0.0035 (7)	−0.0013 (6)	0.0014 (7)
C2	0.0307 (8)	0.0271 (9)	0.0251 (8)	−0.0096 (7)	−0.0017 (7)	0.0035 (7)
C3	0.0269 (8)	0.0295 (9)	0.0235 (8)	−0.0107 (7)	−0.0004 (6)	−0.0004 (7)
C4	0.0273 (8)	0.0291 (9)	0.0240 (8)	−0.0074 (7)	−0.0044 (6)	0.0036 (7)
C5	0.0238 (7)	0.0234 (8)	0.0297 (9)	−0.0061 (6)	−0.0010 (6)	0.0005 (7)
C6	0.0278 (8)	0.0313 (9)	0.0291 (9)	−0.0088 (7)	−0.0048 (7)	−0.0043 (7)
C7	0.0254 (8)	0.0403 (11)	0.0236 (8)	0.0001 (7)	−0.0024 (7)	0.0019 (7)
C8	0.0425 (10)	0.0416 (11)	0.0259 (9)	−0.0241 (9)	−0.0008 (8)	−0.0013 (8)
C9	0.0279 (8)	0.0256 (9)	0.0351 (10)	−0.0081 (7)	0.0020 (7)	−0.0005 (7)
C10	0.0376 (9)	0.0329 (10)	0.0296 (9)	−0.0125 (8)	−0.0077 (7)	−0.0037 (7)
C11	0.0309 (8)	0.0357 (10)	0.0290 (9)	−0.0155 (8)	−0.0047 (7)	−0.0012 (7)
C12	0.0273 (8)	0.0270 (9)	0.0264 (8)	−0.0075 (7)	−0.0049 (7)	0.0010 (7)
C13	0.0361 (9)	0.0311 (10)	0.0319 (9)	−0.0042 (8)	−0.0092 (8)	0.0007 (8)
C14	0.0273 (8)	0.0389 (11)	0.0297 (9)	−0.0056 (8)	−0.0030 (7)	−0.0019 (8)
C15	0.0279 (8)	0.0290 (9)	0.0330 (9)	−0.0106 (7)	−0.0028 (7)	−0.0006 (7)
C16	0.0407 (10)	0.0413 (11)	0.0262 (9)	−0.0067 (9)	−0.0087 (8)	−0.0063 (8)
C17	0.0344 (9)	0.0411 (11)	0.0307 (9)	−0.0032 (8)	−0.0120 (8)	−0.0047 (8)
C18	0.0325 (9)	0.0299 (9)	0.0273 (9)	−0.0069 (7)	−0.0071 (7)	−0.0030 (7)
C20	0.0366 (10)	0.0364 (10)	0.0298 (9)	−0.0055 (8)	−0.0102 (8)	0.0031 (8)
C21	0.0546 (13)	0.0468 (13)	0.0463 (13)	−0.0177 (11)	0.0023 (10)	−0.0065 (10)
C22	0.0651 (15)	0.0489 (14)	0.0455 (13)	−0.0091 (12)	−0.0142 (11)	−0.0077 (11)

### Geometric parameters (Å, º)

**Table:**

Ni1—N5^i^	2.0597 (15)	C1—C6	1.393 (3)
Ni1—N3^ii^	2.0735 (15)	C1—C7	1.516 (2)
Ni1—N1	2.0777 (15)	C2—C3	1.397 (2)
Ni1—O5	2.0904 (12)	C2—H2A	0.9300
Ni1—O1W	2.1048 (12)	C3—C4	1.384 (2)
Ni1—O1	2.1458 (12)	C3—C8	1.513 (2)
S1—O2	1.4516 (14)	C4—C5	1.395 (2)
S1—O4	1.4611 (16)	C4—H4A	0.9300
S1—O1	1.4792 (12)	C5—C6	1.386 (2)
S1—O3	1.4895 (13)	C5—C9	1.506 (2)
N1—C12	1.324 (2)	C6—H6A	0.9300
N1—C11	1.375 (2)	C7—H7A	0.9700
N2—C12	1.344 (2)	C7—H7B	0.9700
N2—C10	1.370 (2)	C8—H8A	0.9700
N2—C7	1.467 (2)	C8—H8B	0.9700
N3—C15	1.325 (2)	C9—H9A	0.9700
N3—C14	1.376 (2)	C9—H9B	0.9700
N3—Ni1^ii^	2.0736 (15)	C10—C11	1.358 (3)
N4—C15	1.343 (2)	C10—H10A	0.9300
N4—C13	1.371 (2)	C11—H11A	0.9300
N4—C8	1.465 (2)	C12—H12A	0.9300
N5—C18	1.322 (2)	C13—C14	1.348 (3)
N5—C17	1.371 (2)	C13—H13A	0.9300
N5—Ni1^i^	2.0597 (15)	C14—H14A	0.9300
N6—C18	1.343 (2)	C15—H15A	0.9300
N6—C16	1.371 (2)	C16—C17	1.355 (3)
N6—C9	1.469 (2)	C16—H16A	0.9300
O1W—H1WA	0.8187	C17—H17A	0.9300
O1W—H1WB	0.8219	C18—H18A	0.9300
O2W—H2WA	0.8318	C20—C20^i^	1.492 (4)
O2W—H2WB	0.8269	C20—H20A	0.9700
O5—C20	1.427 (2)	C20—H20B	0.9700
O5—H1A	0.8129	C21—C22	1.489 (3)
O7—C21	1.420 (3)	C21—H21A	0.9700
O7—H7C	0.8200	C21—H21B	0.9700
O6—C22	1.404 (3)	C22—H22A	0.9700
O6—H6B	0.8200	C22—H22B	0.9700
C1—C2	1.379 (3)		
			
N5^i^—Ni1—N3^ii^	89.38 (6)	C5—C6—H6A	119.9
N5^i^—Ni1—N1	175.70 (6)	C1—C6—H6A	119.9
N3^ii^—Ni1—N1	92.36 (6)	N2—C7—C1	112.19 (14)
N5^i^—Ni1—O5	88.70 (6)	N2—C7—H7A	109.2
N3^ii^—Ni1—O5	176.18 (5)	C1—C7—H7A	109.2
N1—Ni1—O5	89.79 (5)	N2—C7—H7B	109.2
N5^i^—Ni1—O1W	88.85 (6)	C1—C7—H7B	109.2
N3^ii^—Ni1—O1W	93.73 (6)	H7A—C7—H7B	107.9
N1—Ni1—O1W	87.11 (5)	N4—C8—C3	112.00 (15)
O5—Ni1—O1W	89.54 (5)	N4—C8—H8A	109.2
N5^i^—Ni1—O1	93.10 (6)	C3—C8—H8A	109.2
N3^ii^—Ni1—O1	90.67 (5)	N4—C8—H8B	109.2
N1—Ni1—O1	90.82 (5)	C3—C8—H8B	109.2
O5—Ni1—O1	86.13 (5)	H8A—C8—H8B	107.9
O1W—Ni1—O1	175.21 (5)	N6—C9—C5	111.96 (14)
O2—S1—O4	111.46 (11)	N6—C9—H9A	109.2
O2—S1—O1	109.44 (9)	C5—C9—H9A	109.2
O4—S1—O1	110.26 (9)	N6—C9—H9B	109.2
O2—S1—O3	109.46 (9)	C5—C9—H9B	109.2
O4—S1—O3	107.10 (10)	H9A—C9—H9B	107.9
O1—S1—O3	109.06 (7)	C11—C10—N2	105.93 (16)
C12—N1—C11	105.32 (15)	C11—C10—H10A	127.0
C12—N1—Ni1	127.89 (12)	N2—C10—H10A	127.0
C11—N1—Ni1	126.79 (12)	C10—C11—N1	109.93 (16)
C12—N2—C10	107.46 (14)	C10—C11—H11A	125.0
C12—N2—C7	126.22 (16)	N1—C11—H11A	125.0
C10—N2—C7	126.31 (16)	N1—C12—N2	111.36 (15)
C15—N3—C14	105.22 (15)	N1—C12—H12A	124.3
C15—N3—Ni1^ii^	126.02 (12)	N2—C12—H12A	124.3
C14—N3—Ni1^ii^	128.61 (12)	C14—C13—N4	106.18 (16)
C15—N4—C13	107.33 (15)	C14—C13—H13A	126.9
C15—N4—C8	125.92 (16)	N4—C13—H13A	126.9
C13—N4—C8	126.61 (16)	C13—C14—N3	110.02 (16)
C18—N5—C17	105.44 (15)	C13—C14—H14A	125.0
C18—N5—Ni1^i^	128.44 (12)	N3—C14—H14A	125.0
C17—N5—Ni1^i^	126.04 (12)	N3—C15—N4	111.25 (16)
C18—N6—C16	107.12 (15)	N3—C15—H15A	124.4
C18—N6—C9	126.52 (15)	N4—C15—H15A	124.4
C16—N6—C9	126.24 (15)	C17—C16—N6	106.14 (16)
S1—O1—Ni1	138.40 (8)	C17—C16—H16A	126.9
Ni1—O1W—H1WA	118.1	N6—C16—H16A	126.9
Ni1—O1W—H1WB	126.3	C16—C17—N5	109.86 (16)
H1WA—O1W—H1WB	109.6	C16—C17—H17A	125.1
H2WA—O2W—H2WB	108.8	N5—C17—H17A	125.1
C20—O5—Ni1	131.31 (12)	N5—C18—N6	111.43 (15)
C20—O5—H1A	110.7	N5—C18—H18A	124.3
Ni1—O5—H1A	106.8	N6—C18—H18A	124.3
C21—O7—H7C	109.5	O5—C20—C20^i^	108.89 (19)
C22—O6—H6B	109.5	O5—C20—H20A	109.9
C2—C1—C6	119.90 (16)	C20^i^—C20—H20A	109.9
C2—C1—C7	119.62 (16)	O5—C20—H20B	109.9
C6—C1—C7	120.47 (16)	C20^i^—C20—H20B	109.9
C1—C2—C3	120.49 (16)	H20A—C20—H20B	108.3
C1—C2—H2A	119.8	O7—C21—C22	109.84 (19)
C3—C2—H2A	119.8	O7—C21—H21A	109.7
C4—C3—C2	119.28 (16)	C22—C21—H21A	109.7
C4—C3—C8	120.40 (16)	O7—C21—H21B	109.7
C2—C3—C8	120.29 (16)	C22—C21—H21B	109.7
C3—C4—C5	120.59 (16)	H21A—C21—H21B	108.2
C3—C4—H4A	119.7	O6—C22—C21	113.0 (2)
C5—C4—H4A	119.7	O6—C22—H22A	109.0
C6—C5—C4	119.54 (16)	C21—C22—H22A	109.0
C6—C5—C9	120.37 (16)	O6—C22—H22B	109.0
C4—C5—C9	120.09 (16)	C21—C22—H22B	109.0
C5—C6—C1	120.19 (16)	H22A—C22—H22B	107.8
			
O2—S1—O1—Ni1	−139.62 (13)	C7—N2—C10—C11	−178.79 (16)
O4—S1—O1—Ni1	97.44 (15)	N2—C10—C11—N1	−0.3 (2)
O3—S1—O1—Ni1	−19.90 (16)	C12—N1—C11—C10	0.3 (2)
C6—C1—C2—C3	−0.9 (3)	Ni1—N1—C11—C10	−179.26 (12)
C7—C1—C2—C3	−179.63 (15)	C11—N1—C12—N2	−0.27 (19)
C1—C2—C3—C4	1.4 (3)	Ni1—N1—C12—N2	179.31 (11)
C1—C2—C3—C8	179.31 (16)	C10—N2—C12—N1	0.12 (19)
C2—C3—C4—C5	−1.2 (3)	C7—N2—C12—N1	179.00 (15)
C8—C3—C4—C5	−179.09 (16)	C15—N4—C13—C14	−0.6 (2)
C3—C4—C5—C6	0.5 (3)	C8—N4—C13—C14	−176.31 (17)
C3—C4—C5—C9	−178.73 (15)	N4—C13—C14—N3	0.7 (2)
C4—C5—C6—C1	0.1 (3)	C15—N3—C14—C13	−0.5 (2)
C9—C5—C6—C1	179.27 (15)	Ni1^ii^—N3—C14—C13	175.22 (13)
C2—C1—C6—C5	0.2 (3)	C14—N3—C15—N4	0.1 (2)
C7—C1—C6—C5	178.86 (15)	Ni1^ii^—N3—C15—N4	−175.73 (11)
C12—N2—C7—C1	−92.6 (2)	C13—N4—C15—N3	0.3 (2)
C10—N2—C7—C1	86.1 (2)	C8—N4—C15—N3	176.05 (16)
C2—C1—C7—N2	−82.8 (2)	C18—N6—C16—C17	0.8 (2)
C6—C1—C7—N2	98.5 (2)	C9—N6—C16—C17	177.19 (17)
C15—N4—C8—C3	−92.2 (2)	N6—C16—C17—N5	−0.7 (2)
C13—N4—C8—C3	82.8 (2)	C18—N5—C17—C16	0.4 (2)
C4—C3—C8—N4	−129.77 (17)	Ni1^i^—N5—C17—C16	177.31 (13)
C2—C3—C8—N4	52.4 (2)	C17—N5—C18—N6	0.1 (2)
C18—N6—C9—C5	85.2 (2)	Ni1^i^—N5—C18—N6	−176.68 (12)
C16—N6—C9—C5	−90.5 (2)	C16—N6—C18—N5	−0.6 (2)
C6—C5—C9—N6	−97.28 (19)	C9—N6—C18—N5	−176.97 (15)
C4—C5—C9—N6	81.9 (2)	Ni1—O5—C20—C20^i^	99.0 (2)
C12—N2—C10—C11	0.09 (19)	O7—C21—C22—O6	−64.0 (3)

Symmetry codes: (i) −*x*, −*y*+1, −*z*+1; (ii) −*x*, −*y*, −*z*+1.

### Hydrogen-bond geometry (Å, º)

**Table:**

*D*—H···*A*	*D*—H	H···*A*	*D*···*A*	*D*—H···*A*
O1*W*—H1*WA*···O6^iii^	0.82	1.93	2.743 (2)	172
O1*W*—H1*WB*···O2^iii^	0.82	1.88	2.6995 (19)	178
O2*W*—H2*WA*···O3^iv^	0.83	2.00	2.827 (2)	176
O2*W*—H2*WB*···O3	0.83	2.13	2.928 (2)	163
O5—H1*A*···O3	0.81	1.81	2.6168 (18)	169
O7—H7*C*···O4^iv^	0.82	2.07	2.884 (3)	174

Symmetry codes: (iii) *x*−1, *y*, *z*; (iv) −*x*+1, −*y*+1, −*z*+1.

## References

[bb1] Carlucci, L., Ciani, G., Maggini, S. & Proserpio, D. M. (2008). *CrystEngComm*, **10**, 1191–1203.

[bb2] Du, M., Li, C.-P., Chen, M., Ge, Z.-W., Wang, X., Wang, L. & Liu, C.-S. (2014). *J. Am. Chem. Soc.* **136**, 10906–10909. 10.1021/ja506357n 25019403

[bb3] Fan, J., Sun, W.-Y., Okamura, T., Tang, W.-X. & Ueyama, N. (2003). *Inorg. Chem.* **42**, 3168–3175. 10.1021/ic0206847 12739955

[bb4] Hua, Q., Su, Z., Zhao, Y., Okamura, T., Xu, G.-C., Sun, W.-Y. & Ueyama, N. (2010). *Inorg. Chim. Acta*, **363**, 3550–3557.

[bb5] Jacobson, R. (1998). *REQAB*. Molecular Structure Corporation, The Woodlands, Texas, USA.

[bb6] Jiang, H.-L., Liu, B., Lan, Y.-Q., Kuratani, K., Akita, T., Shioyama, H., Zong, F. & Xu, Q. (2011). *J. Am. Chem. Soc.* **133**, 11854–11857. 10.1021/ja203184k 21751788

[bb7] Pan, L., Sander, M.-B., Huang, X.-Y., Li, J., Smith, M., Bittner, E., Bockrath, B. & Johnson, J.-K. (2004). *J. Am. Chem. Soc.* **126**, 1308–1309. 10.1021/ja0392871 14759166

[bb8] Rigaku (2007). *CrystalClear*. Rigaku Corporation, Tokyo, Japan.

[bb9] Sheldrick, G. M. (2008). *Acta Cryst.* A**64**, 112–122. 10.1107/S0108767307043930 18156677

[bb10] Sheldrick, G. M. (2015*a*). *Acta Cryst.* A**71**, 3–8.

[bb11] Sheldrick, G. M. (2015*b*). *Acta Cryst.* C**71**, 3–8.

[bb12] Su, Z., Wang, Z.-B. & Sun, W.-Y. (2010). *Inorg. Chem. Commun.* **13**, 1278–1280.

[bb13] Wan, S.-Y., Huang, Y.-T., Li, Y.-Z. & Sun, W.-Y. (2004). *Microporous Mesoporous Mater.* **73**, 101–108.

[bb14] Xu, G.-C., Ding, Y.-J., Okamura, T., Huang, Y.-Q., Bai, Z.-S., Hua, Q., Liu, G.-X., Sun, W.-Y. & Ueyama, N. (2009). *Cryst. Growth Des.* **9**, 395–403.

[bb15] Zhao, W., Fan, J., Okamura, T., Sun, W.-Y. & Ueyama, N. (2004). *J. Solid State Chem.* **177**, 2358–2365.

[bb16] Zhong, K.-L. (2014). *Acta Cryst.* C**70**, 189–193. 10.1107/S2053229613034347 24508967

